# Neural Responses to Reward and Punishment Stimuli in Depressed Status Individuals and Their Effects on Cognitive Activities

**DOI:** 10.3389/fpsyg.2021.808341

**Published:** 2022-02-07

**Authors:** Yutong Li, Xizi Cheng, Yahong Li, Xue Sui

**Affiliations:** School of Psychology, Liaoning Normal University, Dalian, China

**Keywords:** depression, reward and punishment, event-related potential, cognitive activity, emotion-context insensitivity

## Abstract

Individuals in depressed status respond abnormally to reward stimuli, but the neural processes involved remain unclear. Whether this neural response affects subsequent cognitive processing activities remains to be explored. In the current study, participants, screened as depressed status individuals and healthy individuals by Beck Depression Inventory and Hospital Anxiety Depression Scale, performed both a door task and a cognitive task. Specifically, in each trial, they selected one from two identical doors based on the expectations of rewards and punishments and received the rewarded or punished feedback, and then they performed a cognitive task in which they judged the correctness of a math equation. The neural responses of their choice in the door task were recorded. The results showed that when the two groups received punished feedback, their accuracy was significantly higher than they received rewarded feedback. Compared with the healthy group, the depressed status group spent more time completing cognitive tasks. Analysis of electroencephalography (EEG) data showed that the amplitude of RewP induced by rewarded feedback was larger than that induced by punished feedback, and the amplitude of RewP and fb-P3 induced by the depressed status group was smaller than that of the healthy group. The results of an order analysis showed that the main effects of group variable in fb-P3 and RewP appeared in the second half of the data, and the main effect of feedback type in RewP appeared in the first half of the data. The results showed that the neural response of individuals in depressed status to reward and punishment stimuli was weakened compared with healthy individuals and affected the subsequent cognitive processing to some extent. The effect of feedback appeared in the early stage and gradually decreased. The neural response of individuals in depressed status had a cumulative effect, and the differences appeared in the later stage. The results of this study support the emotional situation insensitive hypothesis, that is, individuals in depressed status are less sensitive to reward and punishment than healthy individuals.

## Introduction

Depression is a poor psychological state. Depressed individuals usually present with low mood, decreased interest, and anhedonia. Depression has a slow progression from mild to major. Individuals with different levels of depression have a common manifestation of abnormal responses to reward and punishment stimuli (Foti et al., 2014; Brush et al., 2018; Klawohn et al., 2021). Depressed individuals were found to be less responsive to reward stimuli than healthy individuals (Henriques and Davidson, 2000; Pizzagalli et al., 2005, 2008). They were less likely to avoid punitive stimuli. Once depressed individuals focus on negative stimuli, it is difficult for them to shift their attention away (Suslow et al., 2020). For example, when viewing emotional images, depressed individuals looked at negative stimuli for longer time than healthy individuals (Duque and Vazquez, 2015; Klawohn et al., 2020).

To explain the abnormal emotional response of depressed individuals to reward and punishment, researchers proposed the positive attenuation hypothesis (Bylsma et al., 2008). The hypothesis holds that depressed individuals are less emotionally responsive to positive stimuli. For example, watching funny movie clips or pleasant scenes was associated with lower positive emotions in depressed subjects than in healthy subjects (Rottenberg et al., 2002). This hypothesis is supported by several studies (Sloan et al., 1997, 2001; Allen et al., 1999). Some researchers also proposed the negative potentiation hypothesis, believing that depressed individuals have stronger emotional responses to negative stimuli (Bylsma et al., 2008). The researchers found that depressed individuals had a stronger electrodermal reactivity to negative social scenarios than healthy individuals (Sigmon and Nelson-Gray, 1992), which is consistent with the negative potentiation hypothesis. The increased response of depressed individuals to negative stimuli may be because the negative cognitive structure of depressed individuals is more sensitive to negative stimuli, leading to enhanced emotional responses to negative stimuli (Beck, 1976). Researchers also proposed the emotion context-insensitivity hypothesis (Rottenberg and Gotlib, 2004). According to this hypothesis, depressed individuals lack the generalization ability to emotional responses, that is, they lack situational adaptive responses to events that cause positive and negative emotions. Studies have found that depressed individuals have a low level of entertainment for the tragic and comic films (Rottenberg et al., 2002), and their emotional responses lack adaptability (Rottenberg and Hindash, 2015).

The above theories explain the abnormal emotional response of depressed individuals to reward and punishment stimuli from different perspectives, and the neural activities behind the abnormal emotional response have attracted researchers’ attention. Studies have found that reward and punishment feedback induce the reward positivity (RewP) component (Proudfit, 2015), which is a relative positivity over frontocentral areas occurring approximately between 250 and 350 ms, and the amplitude of RewP generated in the gain condition is larger than that in loss condition (Proudfit et al., 2015). Compared with healthy individuals, depressed individuals produce a smaller amplitude of RewP (Foti and Hajcak, 2009; Liu et al., 2014; Klawohn et al., 2021). It was also found that the amplitude of RewP was closely related to the severity of depressive symptoms (Bress and Hajcak, 2013; Foti et al., 2014). In addition, the researchers found another electroencephalography (EEG) component, feedback-P3 (fb-P3), associated with responses to reward and punishment stimuli in depressed individuals (Ait Oumeziane et al., 2019; Zhang et al., 2020). The fb-P3 is a positivity peaking between 300 and 600 ms after the onset of the stimulus, with the maximum amplitude at the parietal region. The smaller amplitude of fb-P3 means that individuals allocate fewer attention resources to task-related, infrequent or unexpected stimuli (Courchesne et al., 1975; Donchin and Coles, 1988). The fb-P3 is also related to the salience of motivation in the feedback process (Nieuwenhuis et al., 2005). The greater the motivation, the greater the fb-P3 amplitude. Some studies have found that depressed individuals have small fb-P3 amplitude in response to both gain and loss feedback, and the amplitude gradually decreases with the development of depression (Luking et al., 2021). However, no difference in the amplitude of RewP and fb-P3 between depressed and healthy individuals was found in studies using the monetary incentive delay task paradigm (Landes et al., 2018). In other words, whether RewP and fb-P3 components are abnormal neural responses to reward and punishment stimuli in depressed individuals remains to be further verified.

The current study first focused on whether individuals in depressed status have abnormal neural responses to reward and punishment feedback on their behavior. The second focus was whether their neural response will affect subsequent cognitive processing activities. To this end, this study conducted reward and punishment feedback on the subjects’ choice behavior and recorded the neural responses of the subjects when they saw the feedback stimulus. After the feedback stimulus, the cognitive processing task was set up to explore the influence of the neural response generated by the feedback stimulus on the subsequent cognitive processing. In this study, it was expected that the neural response to reward and punishment stimuli may reduce in depressed status individuals compared with healthy individuals, as reflected in the decreased amplitude of RewP and fb-P3. And this neural response may interfere with the subsequent cognitive processing activities of depressed status individuals. This study provides new experimental evidence for how depressed status individuals respond to feedback on their behavior and also provides differences in cognitive task completion after such neural responses compared with healthy individuals. It can also provide new evidence for improving the explanatory power of relevant theories.

## Materials and Methods

### Participants

To ensure sufficient statistical power, we calculated the required sample size by a power analysis based on the predicted effect size using G* Power 3.1 (Faul et al., 2009). This study was a mixed experiment design. We predicted a medium effect size (*f* = 0.25). With 95% actual power at the 0.05 significance level, the required sample size was 34 individuals. Thirty-five participants were recruited from Liaoning Normal University based on their scores on the Hospital Anxiety Depression scale (HAD) (Barczak et al., 1988) and the Beck Depression Inventory (BDI) (Beck et al., 1961). Participants were included in the depressed status group if they scored equal or higher than 13 on the BDI for depressive symptoms in the past two weeks, and scored equal or higher than 11 on the HAD for depressive symptoms in the past one month. Participants who had scores lower than 4 in the BDI and scores not higher than 7 of HAD were included in the healthy control group. All participants spoke Chinese as their first language and were right-handed as determined by Edinburgh handedness inventory (Oldfield, 1971). All had a normal and corrected-to-normal vision. None of them reported a history of neurological impairments, seizure disorder, and current alcohol. One participant in the depressed status group was excluded due to the self-reported use of psychoactive drugs for depression. Two participants in the depressed status group and one participant in the healthy control group were excluded due to excessive eye movements or EEG artifacts, leaving a final 14 participants (12 males, mean age = 23.46 years, SD = 3.62) in the depressed status group and 17 participants (12 males, mean age = 23.46 years, SD = 3.62) in the healthy control group for the data analysis. Participants submitted written informed consent before the experiment. The Institutional Review Board at Liaoning Normal University approved this experiment and the research work was carried out in accordance with the Declaration of Helsinki.

### Procedure

The participants were asked to perform the door task and the cognitive processing task. These two tasks were administered with the E-prime software v2.0 (Psychology Software Tools, Inc.). It consisted of three blocks of 20 trials. In each trial, the door task was completed first. Two identical images of doors were presented, and participants were asked to select the left or right door by clicking the left or right mouse button, respectively. After the participants made their choice, a gray arrow pointing upward or downward was presented. Participants were informed that they could either win ¥1.00 with the arrow pointing upward or lose ¥0.50 with an arrow pointing downward on each trial (Klawohn et al., 2021), in which they would try to win as much money as possible. Subsequently, the participants completed the cognitive processing task at the end of the trial in which they would judge the correctness of a math equation presented on the center of the screen by pressing the mouse buttons (left/right button press for yes/no responses, and it is counterbalanced across participants). Therefore, the tasks of participants were selected one of two identical doors based on their expectation for reward, then received feedback randomly, and finally completed the judgment of a math equation as quickly and accurately as possible in each trial.

**FIGURE 1:**
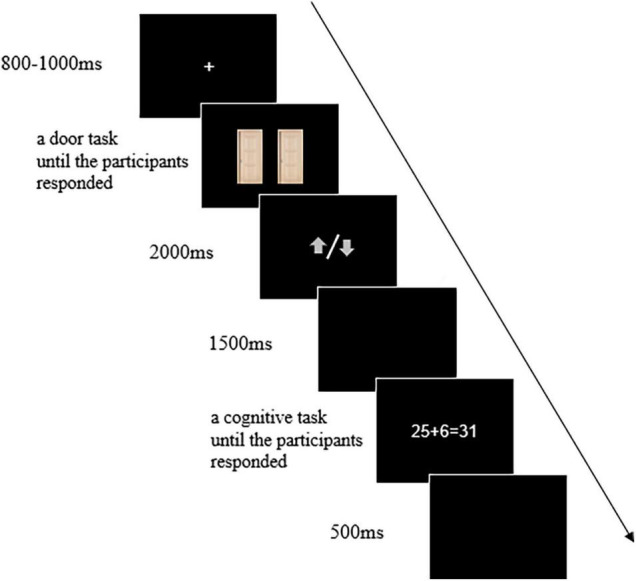
The example for procedure of a trial. Participants selected the left or right door by clicking on the left or right mouse button based on their expectations for the reward, after receiving the feedback performed the cognitive task to judge the correctness of the math equation.

As shown in Figure 1, each trial began with a fixation cross displayed for 800–1000 ms jitters duration, and then the images of two identical doors were presented for 2000 ms which were visible until participants made a choice. After that, the feedback stimulus was presented for 2000 ms. Gain feedback was indicated by a gray arrow pointing upward, while loss feedback was indicated by a gray arrow pointing downward. A blank screen was presented for 1500 ms, followed by the math equation. Participants were asked to judge the equation’s correctness. The math equations were all two digits plus/minus one digit. Across the 60 trials, both gain and loss feedback were equally frequent and presented pseudo-randomly.

### EEG Recording and Analysis

Electroencephalography (EEG) was recorded by a 64-Channel Brain Products system. A reference electrode was located at FCz, and the ground electrode was located at FPz. To monitor eye blinks and movements, two electrodes placed on the supra- and infra-orbital ridges of the right eye were to measure the vertical electrooculogram (EOG), while one electrode placed at the outer canthus of the left eye was to record the horizontal EOG. All electrode impedance was kept below 5 kΩ.

Raw EEG data were processed offline using Brain Vision Analyzer version 2.0 (Brain Products, GmbH; Gilching, Germany). For the data analysis, data were re-referenced to linked mastoids (TP9 and TP10), sampled at 1000 Hz, and filtered by 0.1 Hz high-pass, 30 Hz low-pass filters (slope 24 dB/oct). Additionally, automatic artifact detection was performed to eliminate epochs with a voltage difference of more than 50 μV between sample points, a voltage difference exceeding 200 μV within a trial, or a maximum voltage difference less than 0.5 μV within 100 intervals. After artifact correction, epochs were extracted for feedback stimulus (–200 to 800 ms), which would be corrected with the baseline of 200 ms pre-stimulus interval. For each participant, cleaned epochs were averaged across trials separately for gain and loss feedback conditions. Finally, the mean numbers of used trials were 28.43 (SD = 1.22) for the gain/depressed status condition, 28.97 (SD = 1.31) for the loss/depressed status condition, 29.41 (SD = 1.00) for the gain/control condition, and 29.11 (SD = 0.93) for the loss/control condition.

Based on previous literature, the RewP (250–350 ms) over a mid-frontocentral area (FCz, FC1, FC2, Fz, Cz), fb-P3 (330–430 ms) over a mid-centroparietal area (CP1, CP2, Pz, Cz) were chosen for statistical analysis, which corresponded to the typical latency range and the distribution of the RewP (Chang et al., 2020; Klawohn et al., 2020) and fb-P3 (Wang et al., 2020) components. The mean amplitude of the RewP was calculated by the arithmetic average at electrodes sites in the mid-frontocentral area within a time window of 250–350 ms post feedback onset. The mean amplitude of the fb-P3 was calculated by the arithmetic average, also at electrodes sites in the mid-centroparietal area, within a time window of 330–430 ms post feedback onset. Also, we calculated the peak amplitude of the RewP and fb-P3 in the time window over the interest areas, respectively.

## Results

In order to investigate the neural response of individuals in depressed status to reward and punishment stimuli and its influence on subsequent cognitive processing, 2(group: depressed status vs. healthy control) × 2 (feedback type: gain vs. loss), two factor mixed measures ANOVAs were performed on behavioral and ERP measurements separately. All statistical analyses were performed in SPSS v22 (IBM, Armonk, NY) with an alpha level of 0.05. Significant interactions were analyzed through a simple effects model.

### Behavioral Results for the Cognitive Task

We analyzed the mean accuracy and reaction time for the cognitive task. The behavioral data in each condition was presented in Table 1. With regard to accuracy, the ANOVA results revealed only a significant main effect of feedback type, *F* (1, 29) = 298.15, *p* < 0.001, $\eta_{p}^{2}$ = 0.91. The accuracy in the loss feedback was higher than that in the gain feedback. There was no significant main effect of group, *F* (1, 29) = 0.12, *p* = 0.74. The interaction between feedback type and group was not significant, *F* (1, 29) = 0.26, *p* = 0.61.

**TABLE 1:** The mean accuracies and response times for stimulus types.

	Depressed status group		Healthy control group	
	Gain	Loss	Gain	Loss
RT (ms)	3650 (2272)	3847 (2494)	1921 (329)	1989 (379)
ACC	0.70 (0.06)	0.90 (0.03)	0.71 (0.05)	0.90 (0.07)

*RT = response time; ACC = accuracy, which was defined as the percentage of correct responses out of the total number of trials in each condition; the data in parentheses was standard deviation.*

The reaction time was excluded for those greater or less than 2.5 standard deviations. The ANOVA on the reaction time showed a significant main effect of group, *F* (1, 29) = 9.60, *p* = 0.004, $\eta_{p}^{2}$ = 0.25, in which the depressed status group needs more time to make responses than the healthy control group. However, the significant main effect of feedback type was not found, *F* (1, 29) = 2.82, *p* = 0.10, and the interaction between the feedback type and group was not significant, *F* (1, 29) = 0.66, *p* = 0.42.

### Electrophysiological Results

**FIGURE 2:**
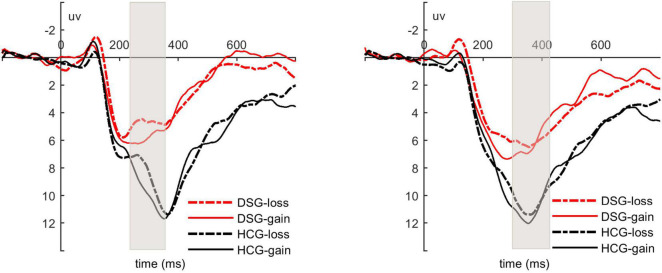
**Left panel:** Grand average waveforms for RewP averaged across the electrode sites of FCz, FC1, FC2, Fz, Cz; **Right panel:** grand average waveforms for fb-P3 averaged across the electrode sites of CP1,CP2, Pz, Cz. DSG = depressed status group. HCG = healthy control group.

We analyzed the mean amplitudes of RewP and fb-P3 evoked by the feedback (see Figure 2 and Table 2). During the RewP time window, the ANOVA results revealed a main effect of feedback type, *F* (1, 29) = 4.73, *p* = 0.038, $\eta_{p}^{2}$ = 0.14. Gain feedback evoked larger RewP amplitudes than loss feedback. The main effect of group reached marginally significant, *F* (1, 29) = 3.41, *p* = 0.075, $\eta_{p}^{2}$ = 0.11, revealing that the depressed status group evoked smaller RewP amplitudes than the healthy control group. However, the interaction between feedback type and group was not significant, *F* (1, 29) = 0.08, *p* = 0.783.

**TABLE 2:** The mean amplitudes (μV) of the ERP components (M ± SE).

	Depressed status group		Healthy control group	
	Gain	Loss	Gain	Loss
RewP	5.73 (1.31)	4.59 (1.47)	10.13 (2.01)	8.66 (1.60)
fb-P3	5.84 (1.40)	6.11 (1.54)	10.90 (1.52)	10.67 (1.83)

During the fb-P3 time window, the ANOVA results revealed a main effect of group, *F* (1, 29) = 4.76, *p* = 0.037, $\eta_{p}^{2}$ = 0.14. The depressed status group evoked smaller fb-P3 amplitudes than the healthy control group. Other significant main effects of feedback type, *F* (1, 29) = 0.001, *p* = 0.97, and the interaction between feedback type and group, *F* (1, 29) = 0.16, *p* = 0.69, were not found.

We also analyzed the peak amplitudes of RewP and fb-P3 evoked by the feedback. For the RewP, the ANOVA results revealed no significant main effect of feedback type, *F* (1, 29) = 0.01, *p* = 0.93, and group, *F* (1, 29) = 3.83, *p* = 0.06. The interaction between feedback type and group was not significant, *F* (1, 29) = 0.14, *p* = 0.71. For the fb-P3, the ANOVA results revealed that there were no significant main effect of feedback type, *F* (1, 29) = 2.17, *p* = 0.15, and group, *F* (1, 29) = 4.12, *p* = 0.52. The significant interaction between feedback type and group was not found, *F* (1, 29) = 0.03, *p* = 0.88.

In order to explore whether the observed effects for the ERP components were different or consistent throughout the whole study, we ran an order analysis by looking at first-half versus second-half data. With regard to the first half of the data, the ANOVA results for the RewP amplitudes revealed a main effect of feedback type, *F* (1, 29) = 4.83, *p* = 0.036, $\eta_{p}^{2}$ = 0.14. Gain feedback evoked larger RewP amplitudes than loss feedback. However, there were no significant main effect of group, *F* (1, 29) = 2.18, *p* = 0.15, and interaction between feedback type and group, *F* (1, 29) = 0.77, *p* = 0.39. For the fb-P3, the ANOVA results showed no significant main effect of feedback type, *F* (1, 29) = 2.72, *p* = 0.11. Also, there were no significant main effect of group, *F* (1, 29) = 2.06, *p* = 0.16, and interaction between feedback type and group, *F* (1, 29) = 2.39, *p* = 0.13.

With regard to the second half of the data, for the RewP, the ANOVA results revealed that only significant main effect of group, *F* (1, 29) = 5.22, *p* = 0.03, $\eta_{p}^{2}$ = 0.15. The depressed status group evoked smaller RewP amplitudes than the healthy control group. The main effect of feedback type, *F* (1, 29) = 0.06, *p* = 0.80, and interaction between feedback type and group, *F* (1, 29) = 0.70, *p* = 0.41, were not significant. For the fb-P3, the ANOVA results showed that main effect of group was significant, *F* (1, 29) = 5.54, *p* = 0.03, $\eta_{p}^{2}$ = 0.16. The depressed status group evoked smaller fb-P3 amplitudes than the healthy control group. However, there were no significant main effect of feedback type, *F* (1, 29) = 0.15, *p* = 0.70, and interaction between feedback type and group, *F* (1, 29) = 0.54, *p* = 0.47.

## Discussion

In this paper, the neural response of individuals in depressed status to reward and punishment feedback was investigated in the same time subsequent cognitive tasks were also investigated. Participants were asked to choose between two doors presented based on their expectations of reward. After the selection, the participants were presented with reward and punishment feedback immediately, and their EEG activities were recorded. After feedback, the math equation was presented and the subjects were asked to judge whether the equation was correct. The results showed that the accuracy rate of the two groups was significantly higher after loss feedback than after gain feedback. The depressed status group took longer time to complete the cognitive tasks than the healthy control group. EEG data analysis showed that the amplitude of RewP induced by gain feedback was larger than that induced by loss feedback. Compared with the healthy control group, the depressed status group produced a lower amplitude of RewP and fb-P3. The order analysis showed that the feedback effect appeared in the first half and adaptation appeared in the second half, which was reflected in the change of RewP amplitude. Group effect appeared in the second half data, which was reflected in the amplitude changes of RewP and fb-P3.

Compared with healthy individuals, individuals with depressed status produced blunted RewP and fb-P3 components in response to reward and punishment stimuli. This insensitivity of neural activity corresponded to poor performance in a subsequent cognitive task, in which the cognitive processing time of the depressed status group was longer than that of the healthy control group. The results showed that individuals in depressed status were less sensitive to both positive and negative events. This is consistent with the emotion context-insensitivity hypothesis (Rottenberg and Hindash, 2015).

EEG results showed that the amplitude of RewP induced by both gain and loss feedback in the depressed status group was smaller than that in the healthy control group, which was consistent with previous research results (Foti and Hajcak, 2009; Bress and Hajcak, 2013; Foti et al., 2014; Kujawa et al., 2014; Liu et al., 2014; Brush et al., 2018; Klawohn et al., 2021). Individuals in depressed status have reduced neural responses to gains and losses and decreased sensitivity to rewards and punishments. The current study also found that the depressed status group induced a smaller amplitude of fb-P3 than the healthy control group. The fb-P3 component is associated with the motivation salience in the feedback process (Nieuwenhuis et al., 2005), and also reflects the allocation of attention to significant stimuli, especially those task-related but infrequent or unexpected stimuli (Courchesne et al., 1975; Donchin and Coles, 1988). This amplitude change indicates that compared with the healthy individuals, the depressed status individuals pay less attention to the stimulus. The reason may well be that compared with the healthy control group, the depressed status group was not sensitive to rewards and punishments in feedback. Feedback did not lead to an increase in attention resources. About the feedback effect that appeared in the first half, the possible reason is that anticipation of reward leads to attention to feedback, which increases the amplitude of RewP. As the experiment progressed, the brain gradually adapted to the feedback, and the brain response weakened, so the feedback effect disappeared. With regard to the electrophysiological response of individuals in depressed status appeared in the second half, the possible reason is that compared with healthy individuals, the responses of an individual in depressed status to reward and punishment feedback become worse and worse, and gradually accumulate, so the differences gradually appear. This brain adaptation to feedback and the accumulation of reduced responses to feedback in depressed status individuals is our speculation that needs to be validated in future studies. Furthermore, individuals in depressed status have reduced neural responses to reward feedback, which corresponds to poor performance in subsequent cognitive task completion. It is likely that a weak neural response leads to poor brain activity, resulting in less preparation for cognitive tasks and longer reaction time. Of course, this slower cognitive activity may also lead to a decreased neural response to the next feedback. However, this study cannot give an exact answer to their specific relationship, which needs to make a further clarification by future studies.

This study also has the following limitations. First, individuals in depressed status were screened only by HAD and BDI scores, i.e., HAD ≥ 11 (HAD range 0-21) and BDI ≥ 13 (BDI range 0–63). However, there are various subtypes in depressed individuals, and some depressed individuals are even a mixture of multiple subtypes. Secondly, this study did not distinguish the various sub-type of depressed subjects, and the sub-type differences of depressed individuals may affect the results of the study. In future studies, we can refine the classification of depressed individuals to eliminate their influence and make the results more accurate.

Combined with the results of this study, it can be concluded that compared with healthy individuals, individuals in depressed status have a weaker neural response to reward and punishment feedback. This weaker neural response corresponded to poor subsequent cognitive processing. This is consistent with the hypothesis of insensitive emotional situations.

## Data Availability Statement

The raw data supporting the conclusions of this article will be made available by the authors, without undue reservation.

## Ethics Statement

The studies involving human participants were reviewed and approved by The Institutional Review Board at Liaoning Normal University. The participants provided their written informed consent to participate in this study.

## Author Contributions

YuL, XC, and XS discussed the research idea, designed the experiment, prepared materials, and performed the experiment. YuL analyzed the data. YuL and XC wrote the manuscript. YaL and XS revised the manuscript. All authors contributed to the article and approved the submitted version.

## Conflict of Interest

The authors declare that the research was conducted in the absence of any commercial or financial relationships that could be construed as a potential conflict of interest.

## Publisher’s Note

All claims expressed in this article are solely those of the authors and do not necessarily represent those of their affiliated organizations, or those of the publisher, the editors and the reviewers. Any product that may be evaluated in this article, or claim that may be made by its manufacturer, is not guaranteed or endorsed by the publisher.
